# Benchmarking the Accuracy of AlphaFold 2 in Loop Structure Prediction

**DOI:** 10.3390/biom12070985

**Published:** 2022-07-14

**Authors:** Amy O. Stevens, Yi He

**Affiliations:** 1 Department of Chemistry and Chemical Biology, University of New Mexico, Albuquerque, NM 87131, USA; ao630@unm.edu; 2 Translational Informatics Division, Department of Internal Medicine, University of New Mexico, Albuquerque, NM 87131, USA

**Keywords:** AlphaFold 2, loop structure prediction

## Abstract

The inhibition of protein–protein interactions is a growing strategy in drug development. In addition to structured regions, many protein loop regions are involved in protein–protein interactions and thus have been identified as potential drug targets. To effectively target such regions, protein structure is critical. Loop structure prediction is a challenging subgroup in the field of protein structure prediction because of the reduced level of conservation in protein sequences compared to the secondary structure elements. AlphaFold 2 has been suggested to be one of the greatest achievements in the field of protein structure prediction. The AlphaFold 2 predicted protein structures near the X-ray resolution in the Critical Assessment of protein Structure Prediction (CASP 14) competition in 2020. The purpose of this work is to survey the performance of AlphaFold 2 in specifically predicting protein loop regions. We have constructed an independent dataset of 31,650 loop regions from 2613 proteins (deposited after the AlphaFold 2 was trained) with both experimentally determined structures and AlphaFold 2 predicted structures. With extensive evaluation using our dataset, the results indicate that AlphaFold 2 is a good predictor of the structure of loop regions, especially for short loop regions. Loops less than 10 residues in length have an average Root Mean Square Deviation (RMSD) of 0.33 Å and an average the Template Modeling score (TM-score) of 0.82. However, we see that as the number of residues in a given loop increases, the accuracy of AlphaFold 2’s prediction decreases. Loops more than 20 residues in length have an average RMSD of 2.04 Å and an average TM-score of 0.55. Such a correlation between accuracy and length of the loop is directly linked to the increase in flexibility. Moreover, AlphaFold 2 does slightly over-predict α-helices and β-strands in proteins.

## 1. Introduction

Protein structure is both critical to understand protein function and key in virtual drug screening and drug design. Technical challenges have held the problem of protein structure prediction at the forefront in biology for the past decade. Traditional experimental methods such as X-ray crystallography and NMR are time consuming and expensive [1]. Furthermore, these experimental methods often produce structures with regions that must be reconstructed to fully understand protein structure and dynamics. Notably, more than half of the proteins in the Protein Data Bank [2] have missing regions [3]. These missing regions most often correspond to loops. Loops are protein regions that are neither α-helices nor β-strands. Because loop regions are most frequently found near the surface of the protein, they are readily exposed to solvent and other proteins [4]. This enables loops to have key roles both in protein structure and biological function [5,6,7]. Structurally, their position allows the loops to shield the proteins’ hydrophobic core; functionally, their position allows loops to be readily involved in protein function and protein–protein interactions [6,7,8]. Because loops are key players in the overall structure and dynamics of proteins [5,9], it is critical to reconstruct missing regions from efforts in protein structure prediction.

Most methods in protein structure prediction heavily rely on the PDB because of the accuracy of homology modeling [10]. Homology modeling builds protein structures by observing sequential patterns for proteins with existing structures. Unlike *α*-helices and *β*-strands, loops have high sequence variability and structural irregularity [11], and thus have a larger deviation from homologue templates [12,13,14,15,16,17]. Lacking an accurate homologue, loops remain the most inaccurate or altogether missing regions of the model [18]. Notably, not all loops are equal when it comes to protein structure prediction. Long loops are even more difficult to reconstruct than short loops because of the nature of the data accessible in the PDB. While it has a reasonable dataset of short loops [19,20], the PDB is missing adequate data to build long loops. Without adequate homologues, ab initio methods are left to predict the structure of long loops. However, ab initio methods have shown to be much less accurate than knowledge-based methods [21].

Various protein loop structure prediction methods have gained the attention of the scientific community, including RosettaNGK [22], GalaxyLoop-PS2 [23], DaReUS-Loop [24], ArchPRED [25], FREAD [26], SuperLooper2 [27], LoopIng [28], CODA [29] and Sphinx [21]. Many of these methods have excelled in community-wide CASP experiments. RosettaNGK is an ab initio method that uses a hybrid energy function that combines both physics-based and knowledge-based energy terms. GalaxyLoop-PS2 is also an ab initio method that implements a unique energy function. GalaxyLoop-PS2 considers multiple energy terms, such as short-range, hydrophobic, and electrostatic interactions. LoopIng is a knowledge-based method that considers both sequence and geometry. Finally, CODA and Sphinx are hybrid loop prediction methods that combine both ab initio and knowledge-based methods. While these protein structure prediction methods have proved to be useful tools, AlphaFold 2 has recently surpassed them all.

The original AlphaFold [30] outperformed all other teams at the Critical Assessment of protein Structure Prediction (CASP 13) competition in 2018. The newly upgraded AlphaFold 2 [30] produced predictions that approach a score of 90 in the global distance test (GDT_TS), where a score above 90 is considered roughly equivalent to the accuracy of an experimentally predicted structure. The purpose of this work is to explore the accuracy of AlphaFold 2 in specifically predicting loop regions of protein structures. Here, we consider a dataset of 31,650 loop regions at least three residues in length from 2613 proteins with both experimentally determined structures and AlphaFold 2 predicted structures. Both RMSD and TM-score indicate that AlphaFold 2 is a good predictor of the structure of loop regions. Furthermore, DSSP analysis indicates that AlphaFold 2 slightly over-predicts regular secondary structure content. Lastly, we see that as the length of the loop increases, the accuracy of predictions by AlphaFold 2 decreases, as indicated by RMSD, TM-score, and DSSP.

## 2. Methods

We build a dataset of 31,650 loop regions at least 3 residues in length from a set of 2613 crystal structures that were deposited in the Protein Data Bank after AlphaFold 2 was trained. Non-secondary structural regions within the crystal structures were identified by DSSP analysis [31], where each amino acid is assigned a specific secondary structure type (none, turn, bend, parallel beta sheet, antiparallel beta sheet, alpha helix, pi helix, or 3–10 helix). Any residue classified as none, turn, or bend was considered as a loop region and included in our further statistical analyses. The corresponding tertiary structures of the loop regions are extracted from both the experimental structure and the AlphaFold 2 structure by using BioPython [32]. All AlphaFold 2 structures were obtained from the AlphaFold Protein Structure Database at https://alphafold.ebi.ac.uk (accessed on 3 January 2022) [30]. Note that the experimental structure and the AlphaFold 2 structure for each loop region are 100% sequence identity. The link to download the dataset used in the work is available at the end of the paper.

## 3. Results

**Figure 1:**
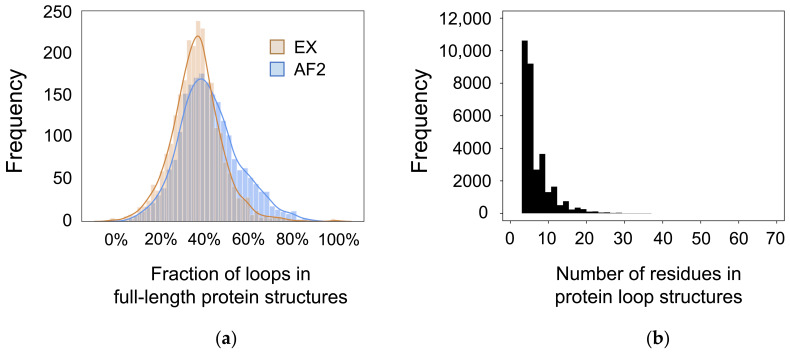
Summary of the dataset. (**a**) Fraction of coiled residues in the full-length protein structures based on DSSP analysis. The tan curve represents DSSP analysis across experimentally determined structures (EX), and the blue curve represents DSSP analysis across AlphaFold 2 predicted structures (AF2). (**b**) Length of each loop structure in the dataset. Loops range from 1 to 65 residues in length.

This work considers a dataset of 2613 proteins with both experimentally determined structures and AlphaFold 2 predicted structures. The fraction of the loop regions in a protein can affect the prediction accuracy of any structure prediction software. To quantify the fraction of loop residues in each protein in our dataset, we performed DSSP [33] analysis across each full-length protein. DSSP, or Dictionary of Secondary Structure of Proteins [33], analysis assigns secondary structure types to each residue in a protein. Each residue is assigned a secondary structure type of either none, turn, bend, parallel beta-sheet, antiparallel beta-sheet, alpha helix, pi helix, or 3–10 helix. Loop residues are defined by the DSSP secondary structure types as none, turn, and bend. As shown in Figure 1, the fraction of loop residues in the experimentally determined structures (EX, tan) and the AlphaFold 2 predicted structures (AF2, blue) and are in relatively good agreement with each other. In both cases, the proteins in the dataset have a Gaussian distribution of the fraction of loop residues centered around 40%. Notably, the AlphaFold 2 predicted structures on average have a slightly greater fraction of loop residues (none, turn, or bend) than the experimentally determined structures. All subsequent analysis is performed only over the loop regions of the given dataset (the 31,650 loops). As shown in Figure 1b, this dataset is comprised of loops ranging from 1 to 65 residues in length. Notably, 82.7% of loops are comprised of fewer than 10 residues, and 98.2% of loops are comprised of fewer than 20 residues.

To quantify the accuracy of AlphaFold 2 in predicting the structure of loop regions, the Root Mean Square Deviation (RMSD) and the Template Modeling score (TM-score) [34] were calculated for each AlphaFold 2 predicted loop structure using the equivalent experimentally predicted loop structure as a reference. RMSD is a traditional metric that has been a standard in comparing protein structures. It measures the average distance between atoms of structurally aligned proteins. Challengingly, RMSD is limited by size-dependency, so it is difficult to directly compare proteins of different sizes with RMSD. TM-score is a metric that was designed to overcome the imperfections of RMSD when comparing protein structures. First, the TM-score weights errors that occur at short distances stronger than those at long distances. Second, TM-score includes a length-dependency term to normalize distance errors. As a result, TM-score produces values that, unlike RMSD, are independent of size. In this work, we will consider both traditional RMSD and size-independent TM-score to explore the accuracy of AlphaFold 2.

**Figure 2:**
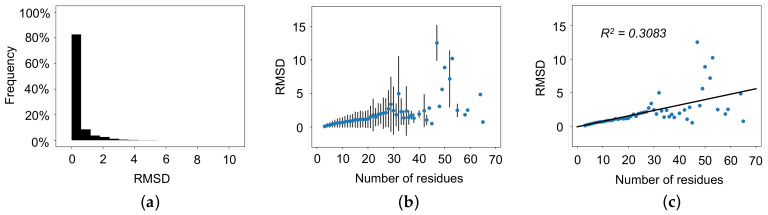
RMSD of AlphaFold 2 predicted structures with the equivalent experimentally determined structure as a template. (**a**) RMSD of all loops region. (**b**) Average RMSD as a function of the number of residues in a loop with standard error bars. (**c**) Average RMSD as a function of the number of residues in a loop with linear regression (R^2^ = 0.3083). RMSD increases as the number of residues in the loop regions increases.

**Figure 3:**
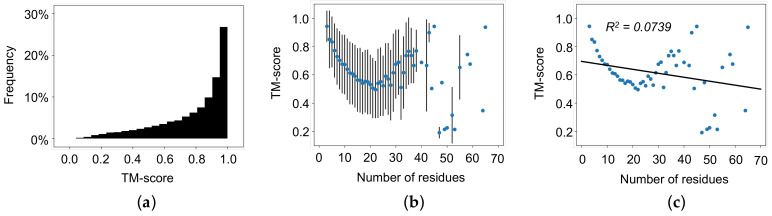
TM-score of AlphaFold 2 predicted structures with the equivalent experimentally determined structure as a template. (**a**) TM-scores of all loop regions. (**b**) Averaged TM-score as a function of the number of residues in a loop with standard error bars. (**c**) Averaged TM-score as a function of the number of residues in a loop with linear regression (R^2^ = 0.0739). TM-score increases as the number of residues in the loop regions increases.

The average RMSD of all loop regions is 0.44 Å, and 89.6% of all loops have an RMSD less than 1.0 Å (Figure 2a). The average TM-score of all loop regions is 0.78, and 86.7% of all loops have a TM-score greater than 0.5 (Figure 3a). Next, we calculated the average RMSD and the average TM-score based on the number of residues in the loop. As shown in Figure 2b, RMSD increases with increasing the length of the loops up to approximately 20 residues. Once the loop length is greater than 30 residues, it is difficult to assess the reliability of the AlphaFold 2’s performance because of the large variation with limited data points. Furthermore, a linear correlation (R^2^ = 0.3083) between the number of residues and RMSD (Figure 2c) was obtained. Different from RMSD, the TM-score weights local structure accuracy. As shown in Figure 3b, TM-score decreases with increasing loop length up to approximately 20 residues. Like RMSD, it is difficult to assess AlphaFold 2’s performance at lengths greater than 20 residues because of the large variation with limited data points. Figure 3c provides the linear correlation (R^2^ = 0.0739) between the number of residues and TM-score across all lengths of loops. It should be noted that the linear regression of average RMSD as a function of the number of residues for loops up to 20 residues in length has a R^2^ value of 0.9648 Å, while the linear regression of average RMSD as a function of the number of residues in a loop for loops greater than 20 residues in length has a R^2^ value of 0.1167. Similarly, the linear regression of average TM-score as a function of the number of residues in a loop for loops up to 20 residues in length has a R^2^ value of 0.8967, while the linear regression of average TM-score as a function of the number of residues in a loop for loops greater than 20 residues in length has a R^2^ value of 0.0045. These results indicate that both RMSD and TM-score indicate good agreement for short loop regions between the AlphaFold 2 predicted structures and the experimentally determined structures. AlphaFold 2 predictions should be carefully evaluated for loop regions consisting of 20 or more residues.

Previous work has suggested that AlphaFold 2 may over-confidently predict structures that correspond to unresolved regions of experimentally determined structures [35]. Here, we used DSSP analysis to determine if AlphaFold 2 has the tendency to over-predict secondary structure content in protein loop regions. We performed DSSP analysis to determine the secondary structure type of each residue in all 31,650 loop regions. It should be noted that full DSSP representation is used for this analysis. After summing the number of residues that correspond to each type across all loop regions, we divided these sums by the total number of residues (212,630 residues in 31,650 loops) to report the fraction of each DSSP secondary structure type. This calculation was performed for both the experimentally determined structures and the AlphaFold 2 predicted structures. Table 1 reports the fraction of each DSSP secondary structure type in the experimental and AlphaFold 2 structures. Our results suggest that AlphaFold 2 has the tendency to slightly over-predict secondary structure content, as 2.28% of residues in loop regions of AlphaFold 2 predicted structures have been assigned a secondary structure type of parallel beta sheet, antiparallel beta sheet, alpha helix, pi helix, or 3–10 helix. Interestingly, our data suggest that AlphaFold 2 specifically over-predicts helices, as 88.5% of predicted secondary structure corresponds to helices (alpha helices, pi helices, or 3–10 helices) while only 11.5% of predicted secondary structure corresponds to beta sheets (parallel beta sheets or antiparallel beta sheets).

**Figure 4:**
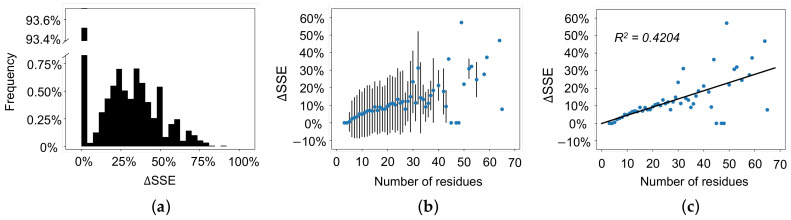
Percent change in secondary structure content (∆SSE) from experimentally determined structures to AlphaFold predicted structures. (**a**) ∆SSE of all loop regions. Greater than 90% of loops have a ∆SSE of 0%. (**b**) Average ∆SSE as a function of the number of residues in a loop with standard error bars. (**c**) Average ∆SSE as a function of the number of residues in a loop with linear regression (R^2^ = 0.4204). ∆SSE increases as the number of residues in the loop regions increases.

While the above DSSP analysis considers all loop regions as a sum, here we will consider secondary structure content changes in each individual loop region. We calculated the percent change in secondary structure content (∆SSE) from any given experimentally determined structure to its corresponding AlphaFold 2 predicted structure. AlphaFold 2 accurately predicts no secondary structure content in greater than 90% of loops (Figure 4a, ∆SSE is equal to 0%). Oppositely, AlphaFold 2 over-predicts the content of secondary structure content in approximately 6.5% of loops (Figure 4a, ∆SSE greater than 0%). Next, we calculated the average percent change in secondary structure content as a function of the number of residues in each loop. Our results indicate that the percent change in secondary structure content increases as the number of residues in the loop increases (Figure 4b). In other words, as the length of the loop increases, AlphaFold 2’s tendency to over-predict the secondary structure content also increases. Our dataset suggests a linear correlation (R^2^ = 0.4204) between the number of residues and ∆SSE (Figure 4c).

**Figure 5:**
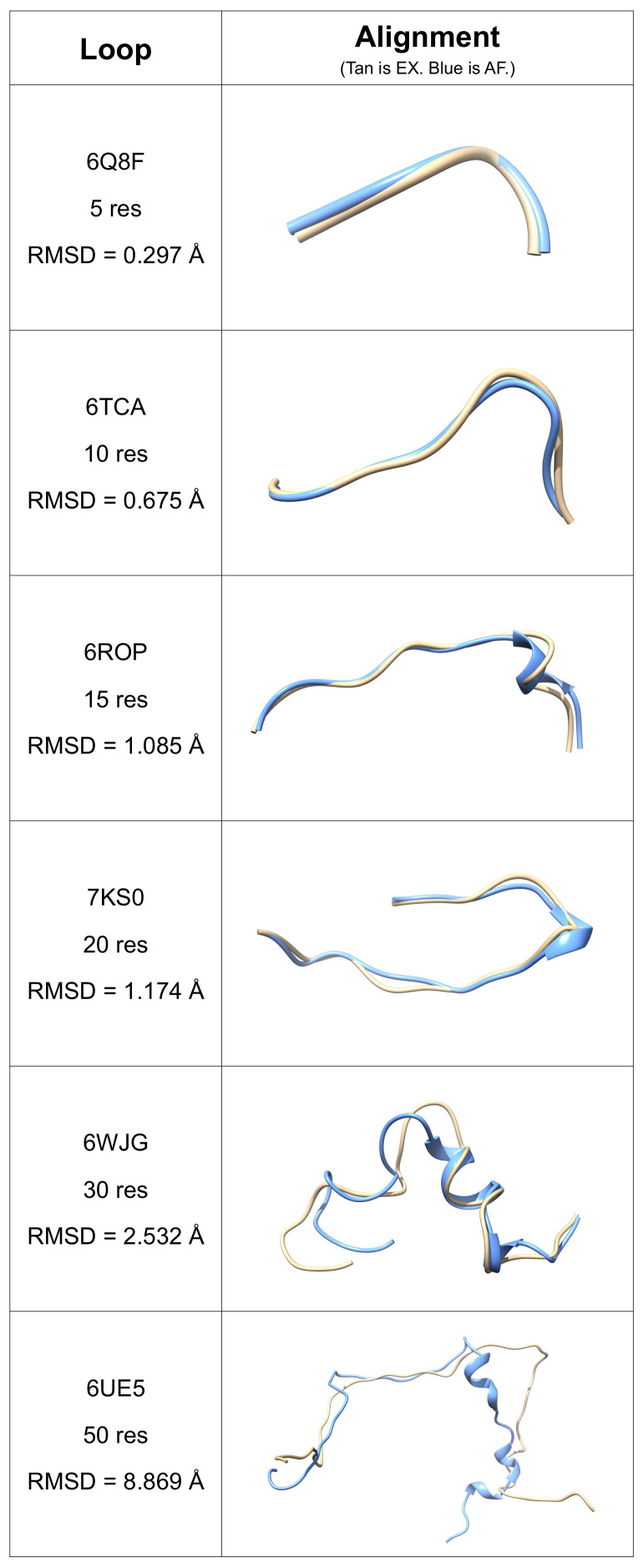
Representative examples of AlphaFold 2 predictions. The experimentally determined structures (EX) are shown in tan, and the AlphaFold 2 predicted structures (AF) are shown in blue. As the number of residues in the loop increases, the RMSD between the experimentally determined structures and the AlphaFold predicted structures also increases. Furthermore, as the number of residues in the loop increases, the secondary structure content in the AlphaFold 2 predicted structures also increases.

We selected representative loops to visualize the alignment of AlphaFold 2 predicted structures to experimentally determined structures. Here, we selected six representative loops from six example proteins: 6Q8F [36] (5 res), 6TCA [37] (10 res), 6ROP [38] (15 res), 7KS0 [39] (20 res), 6WJG [40] (30 res), and 6UE5 [41] (50 res). It is important to note that these examples were selected because they have an RMSD closest to the average RMSD of loops of the given length. The structural alignment of each AlphaFold 2 predicted structure (AF, blue) to its corresponding experimentally determined structure (EX, tan) is shown in Figure 5. Figure 5 provides a visual representation that as the number of residues in the loop increases, the RMSD also increases. It also provides a visual representation that as the number of residues in the loop increases, the secondary structure content increases. As suggested by DSSP analysis, this increase in secondary structure content most prevalently corresponds to helices.

**Figure 6:**
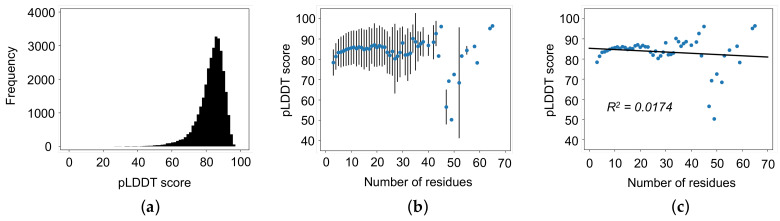
pLDDT scores of AlphaFold 2 predicted structures. (**a**) pLDDT scores of all loop regions. (**b**) Average pLDDT scores as a function of the number of residues in a loop with standard error bars. (**c**) Average pLDDT scores as a function of the number of residues in a loop with linear regression (R^2^ = 0.0174). pLDDT scores are not correlated with the number of residues in the loop regions.

Our dataset indicates that the accuracy of AlphaFold predicted structures decrease as the number of residues in the loop region increases. This correlation between accuracy and length of loop is directly linked to the increase in flexibility. To explore this possibility, we calculated the average pLDDT score of each AlphaFold 2 predicted loop structure by averaging the pLDDT score of each atom in the given loop. The average pLDDT score of all loop regions is 82.9, and 73.2% of all loops have a pLDDT score greater than 80.0 (Figure 6a). Next, we calculated the average pLDDT score based on the number of residues in the loop. As shown in Figure 6b,c, pLDDT score is not clearly correlated with increasing the length of the loops (R^2^ = 0.0174). Interestingly, these results suggest that pLDDT scores are not indicative of the widely accepted belief that increasing loop length will decrease the accuracy of the prediction.

**Figure 7:**
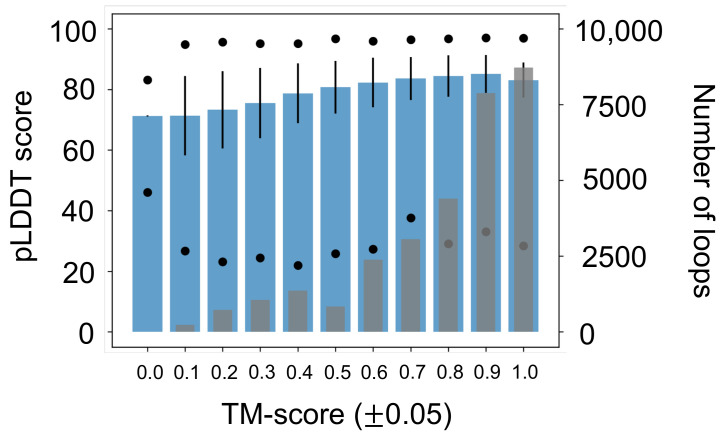
pLDDT score as a function of TM-score (blue) with standard error bars and minimum/maximum pLDDT scores. As TM-score score increases, the average pLDDT score increases. Gray bars represent the number of loops in each TM-score range. Most loops (16,596 or 52.4%) have a TM-score between 0.85 and 1.00.

Subsequently, to understand how well AlphaFold 2 pLDDT scores correspond to the accuracy of the predicted structure, we explored the correlation between pLDDT scores and TM-score. Our calculations show that pLDDT scores increase with increasing TM-score (Figure 7). These results suggest that AlphaFold 2 pLDDT scores properly correspond to the accuracy of the predicted structure. Additional correlation analyses were performed to explore any correlation between b-factor and pLDDT score (Figure S1), the number of residues in each loop and the number of residues in the full-length protein (Figure S2), hydrophilicity and pLDDT score (Figure S3), and hydrophobicity index and pLDDT score (Figure S4). We did not observe any clear correlations. These analyses are included in the SI.

**Figure 8:**
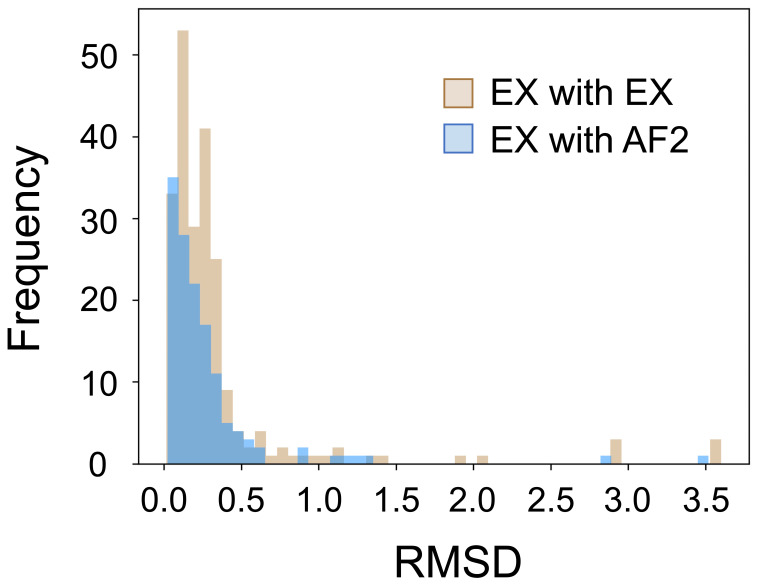
Comparing the experimental range of accuracy to the accuracy of AlphaFold 2 predictions with RMSD. The experimental range of accuracy has an average RMSD of 0.35 Å, and the accuracy of AlphaFold 2 prediction against the experimentally predicted structures has an average RMSD of 0.28 Å.

Lastly, we considered how the structural variability of loops affects predictions by AlphaFold 2. By definition, loops are highly flexible regions that can take multiple structures. Whether experimentally determined or computationally predicted, a loop structure can only represent one of the many possibilities in the loop’s structural ensemble. Because of this, directly comparing the AlphaFold 2 predicted loop structure to an experimentally determined structure has obvious limitations. Our goal was to explore whether the loop regions of AlphaFold 2 predicted structures fall within differences between the experimentally determined structures. We selected five proteins from the dataset with at least three unique experimentally determined structures, all without missing residues. We compared two sets of RMSD values: (1) the RMSD between loops of different experimentally determined structures of one protein and (2) the RMSD between loops of the AlphaFold 2 prediction structure and each experimentally determined structures of the same protein. We repeated these RMSD calculations for all five selected proteins. The first set of RMSD values represent the experimental range of accuracy (Figure 8, tan), and the second set of RMSD values represent the accuracy of AlphaFold 2 predictions against experimentally predicted structures (Figure 8, blue). Results are shown in Figure 8. The experimental range of accuracy has an average RMSD of 0.35 Å, and the accuracy of AlphaFold 2 prediction against the experimentally predicted structures has an average RMSD of 0.28 Å. These results suggest that AlphaFold 2 predicted structures fall within the experimental range of accuracy.

## 4. Discussion

AlphaFold 2 has significantly outperformed all other protein structure prediction methods available today. This work demonstrates that AlphaFold 2 can accurately predict the structure of short loop regions in proteins, especially for loops shorter than 10 residues. However, extra caution is needed when dealing with longer loop regions. Because the vast majority of loop regions in our dataset are short loop regions (98.2% of loops are less than 20 residues in length), AlphaFold 2 predicted structures have an average RMSD of 0.44 Å and an average TM-score of 0.78. Furthermore, AlphaFold 2 predicted structures report no secondary structure content in approximately 93% of loop regions. These results demonstrate that AlphaFold 2 can slightly over predict the regular secondary structures (α-helices and β-strands).

RMSD increases as a function of the length of the loop, as loops comprised of less than 10 residues have an average RMSD of 0.33 Å while loops comprised of more than 20 residues have an average RMSD of 2.04 Å. The TM-score decreases as a function of the length of the loop, as loops comprised of less than 10 residues have an average TM-score of 0.82 while loops comprised of more than 20 residues have an average TM-score of 0.55. Echoing trends in RMSD and TM-score, secondary structure content also increases in AlphaFold 2 predicted structure as the number of residues in the loop region increases. Loops comprised of less than 10 residues have an average ∆SSE of 1.3% while loops comprised of more than 20 residues have an average ∆SSE of 12.5%. In other words, the AlphaFold 2 predicted structures of loops comprised of more than 20 residues have 12.5% more secondary structure content than their corresponding experimentally determined structures.

The correlation between accuracy and length of the loop is linked to the increase in flexibility. The pLDDT score of AlphaFold 2 has been suggested to be an indicator of the flexibility/disorder of a residue/region [30]. In our dataset, average pLDDT scores do not clearly correlate with the length of the loop region. In the case of loop regions, it seems that the average pLDDT scores failed to distinguish the prediction accuracy. Interestingly, the average pLDDT scores do correlate to the accuracy of the prediction based on TM-score, suggesting that AlphaFold 2’s pLDDT scores properly correspond to the accuracy of the predicted structure. In summary, this work supports AlphaFold 2 as a good predictor of the structure of loop regions while exposing limitations of AlphaFold 2 as the length of loop increase.

## Figures and Tables

**Table 1:** Fraction of DSSP secondary structure types in experimentally determined and AlphaFold 2 predicted structures. AlphaFold 2 predicted secondary structure content (parallel beta sheet, antiparallel beta sheet, alpha helix, pi helix, or 3–10 helix) in 2.28% of loop residues.

DSSP Secondary Structure Type	Experimental Structures	AlphaFold 2 Structures
None	78.64%	76.66%
Turn	12.92%	14.51%
Bend	8.44%	6.55%
Parallel beta sheet	0% *	0.10%
Antiparallel beta sheet	0% *	0.16%
Alpha helix	0% *	0.74%
Pi helix	0% *	0.03%
3–10 helix	0% *	1.25%

* By definition, the experimental structures of loop regions in our dataset have 0% parallel beta sheet, antiparallel beta sheet, alpha helix, pi helix, and 3–10 helix.

## Data Availability

The complete loop structure database (containing 31,650 loops) is available to download as a compressed Available online: https://tinyurl.com/3xk5dx3n (accessed on 11 May 2022).

## Supplementary Materials

The following supporting information can be downloaded at: https://www.mdpi.com/article/10.3390/biom12070985/s1, Figure S1: Correlation between b-factor and pLDDT score; Figure S2: Correlation between the number of residues in each loop region and the number of residues in the corresponding full-length protein; Figure S3: Correlation between the fraction of hydrophilic residues and the accuracy of AlphaFold 2 predicitons in loops greater than 20 residues in length; Figure S4: Correlation between the hydrophobicity index and the accuracy of AlphaFold 2 predicitons in loops greater than 20 residues in length.
