# Supplementary material for: Benchmarking the Accuracy of AlphaFold 2 in Loop Structure Prediction
Source: Biomolecules. 2022 Jul 14;12(7):985. doi: 10.3390/biom12070985 (PMC9312937; doi:10.3390/biom12070985)
Supplement: Supplementary file 1 [file biomolecules-12-00985-s001.zip › biomolecules-1772594-supplementary.pdf]

Supplementary information for

Benchmarking the accuracy of AlphaFold 2 in loop structure prediction

Amy O. Stevens [1] and Yi He [1,2]*

[1] Department of Chemistry and Chemical Biology, University of New Mexico, Albuquerque, NM 87131, USA
[2] Translational Informatics Division, Department of Internal Medicine, University of New Mexico, Albuquerque, NM 87131, USA

* Corresponding authoer: Yi He (email: yihe@unm.edu)

SUPPLEMENTAL MATERIAL:

We explored the correlation between b-factor and pLDDT score. Figure S1a plots pLDDT score as a function of b-factor for each loop in the dataset. There is no clear correlation (R^2=0.0056). Next, we calculated the average pLDDT score and the average b-factor for loops of the same length. As shown in Figure S1b, there is also no clear correlation (R^2=0.1571). This is understandable because the pLDDT score corresponds to the model's predicted score on the lDDT-Cα metric, meaning that, even for regions with large fluctuations, as long as sufficient template sequences can be identified, the pLDDT score will still be very high.

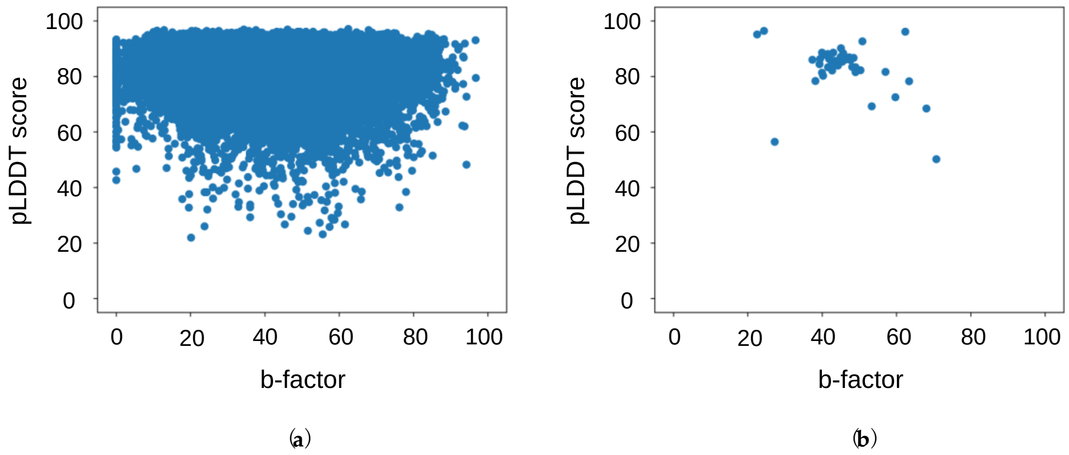

Figure S1. Correlation between b-factor and pLDDT score. (**a**) pLDDT score as a function of b-factor for each loop in the dataset. (**b**) Average pLDDT score as a function of average b-factor for loops with the same number of residues.

To explore the correlation between the number of residues in a loop region and the total number of residues in the corresponding protein, Figure S2a plots the number of residues in the full-length protein as a function of the number of residues in the corresponding loop region. Presumably, a protein with a high percentage of loop regions may have larger fluctuations, thus, more difficult to predict. However, there is no clear correlation (R^2=0.0019). Next, we calculated the average number of residues in the full-length proteins based on the number of residues in the loop. Figure S2b plots the average number of residues in full-length proteins as a function of the number of residues in the loop regions. Again, there is no clear correlation (R^2=0.0442).

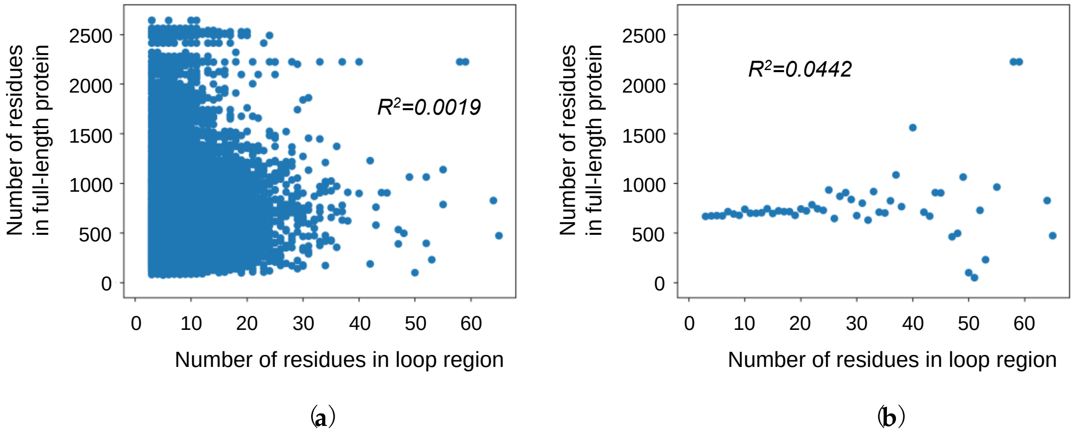

Figure S2. Correlation between the number of residues in each loop region and the number of residues in the corresponding full-length protein. (**a**) Number of residues in the full-length protein as a function of the number of residues in the corresponding loop region. There is no clear correlation (R^2=0.0019). (**b**) Average number of residues in full-length proteins as a function of the number of residues in the loop regions. There is no clear correlation (R^2=0.0442).

RMSD and TM-score analyses suggest that trends in accuracy become unclear in loops greater than 20 residues in length. With this, we performed additional analyses on loops comprised of more than 20 residues to determine if the "type" of long loop affects the accuracy of the AlphaFold 2 prediction. Here, we define loop "types" by the fraction of hydrophilic residues and the hydrophobicity index. First, we consider the fraction of hydrophilic residues in the accuracy of AlphaFold 2 predictions using both RMSD and TM-score. Figure S3a plots RMSD as a function of the fraction of hydrophilic residues across loops greater than 20 residues in length. There is no clear correlation (R^2=0.0007). Figure S3b plots TM-score as a function of the fraction of hydrophilic residues across loops greater than 20 residues in length. There is no clear correlation (R^2=0.0083).

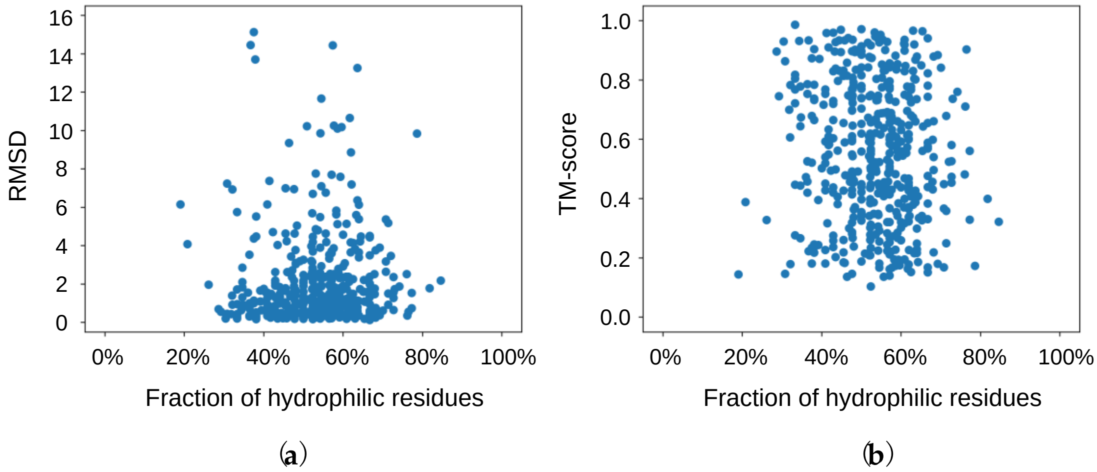

Figure S3. Correlation between the fraction of hydrophilic residues and the accuracy of AlphaFold 2 predictions in loops greater than 20 residues in length. (**a**) RMSD as a function of the fraction of hydrophilic residues across loops greater than 20 residues in length. There is no clear correlation (R^2=0.0007). (**b**) TM-score as a function of the fraction of hydrophilic residues across loops greater than 20 residues in length. There is no clear correlation (R^2=0.0083).

Next, we consider the hydrophobicity index in the accuracy of AlphaFold 2 predictions using both RMSD and TM-score. Figure R4a plots RMSD as a function of the hydrophobicity index across loops greater than 20 residues in length. There is no clear correlation (R^2=0.0001). Figure S4b plots TM-score as a function of the hydrophobicity index across loops greater than 20 residues in length. There is no clear correlation (R^2=0.00002). RMSD and TM-score suggest that the hydrophilicity of loops greater than 20 residues in length is not a significant contributor to the accuracy of the predictions

by AlphaFold 2. It is worth noting that these results may be in part due to the number of loops greater than 20 residues in length in our dataset. 98.5% of loops in our dataset range from 3 to 20 residues in length. Fewer than 500 loops in our dataset have a length greater than 20 residues. Moreover, various lengths (including 38, 40, 42, 43, 44, 45, 46, 48, 49, 50, 52, 53, 55, 58, 59, 64, and 65) have only one or two loop structures available. It is possible that a dataset with more loops at longer lengths may alter these results.

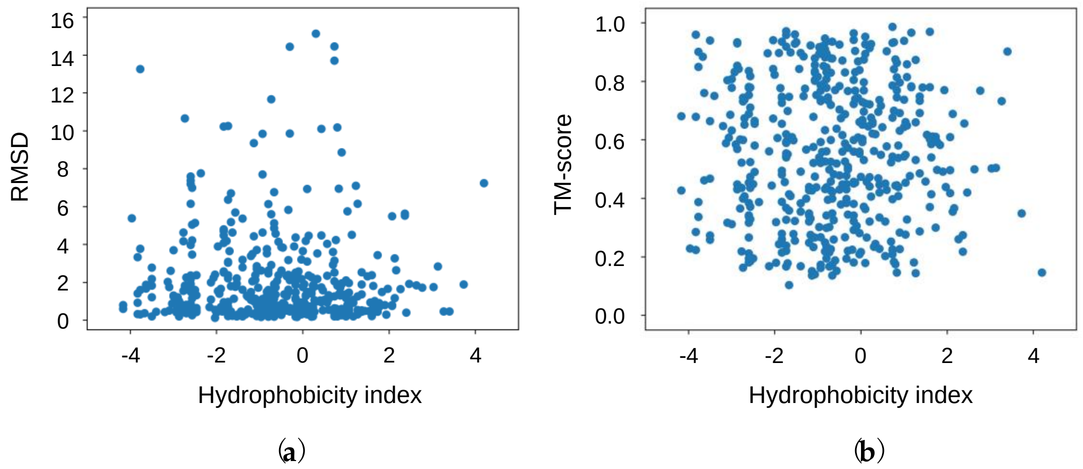

Figure S4. Correlation between the hydrophobicity index and the accuracy of AlphaFold 2 predictions in loops greater than 20 residues in length. (**a**) RMSD as a function of the hydrophobicity index across loops greater than 20 residues in length. There is no clear correlation (R^2=0.0001). (**b**) TM-score as a function of the hydrophobicity index across loops greater than 20 residues in length. There is no clear correlation (R^2=0.00002).
